# The complete mitochondrial genome of the surgeon fish *Acanthurus lineatus* Linnaeus 1758 (Perciformes: Acanthuridae)

**DOI:** 10.1080/23802359.2016.1197061

**Published:** 2016-11-12

**Authors:** Biji Gurupatham Devadhasan, Prakash Vincent Samuel Gnana, Vladimir Benes

**Affiliations:** a Department of Zoology, Nesamony Memorial Christian College, Marthandam, Tamil Nadu, India;; b Centre for Marine Science and Technology, Rajakkamangalam Campus, Manonmaniam Sundaranar University, Rajakkamangalam, Tamil Nadu, India;; c EMBL Heidelberg, Genomics Core Facility, Heidelberg, Germany

**Keywords:** Acanthuridae, *Acanthurus lineatus*, mitochondrial genome, surgeon fish, phylogeny

## Abstract

Acanthurid fishes are highly ornamental coral reef fishes, commonly called as surgeon fishes. In this study, the complete mitochondrial genome of *Acanthurus lineatus* was determined (GenBank accession no EU273284). The genome was circular, double-stranded molecules of 16,532 bp length encoded with 37 mitochondrial genes. It contained 13 protein-coding genes, 22tRNA genes, 2 rRNA genes and 852 bp long control region. The entire nucleotide composition was 29.84% A, 28.07% C, 26.60% T and 15.49% G with an A + T content of 56.44%. Phylogenetic relationship of *A. lineatus* among the closely related species of Perciformes was studied based on H-strand protein-coding genes using the maximum parsimony method.

Acanthuridae is the largest and best known family in the Acanthuroidei comprising surgeon fishes, tangs and unicornfishes found in most tropical and subtropical seas of the world, usually inhabiting coral reefs and sea grass beds (Choat & Bellwood 1991). *Acanthurus* is found throughout the Indian Ocean. Sequencing of *Acanthurus lineatus* mitochondrial genome could provide useful genetic resources to examine the level of genetic diversity population genetics, phylogenetics and taxonomy.

In this present study, the complete mitochondrial genome of *Acanthurus lineatus* was amplified, cloned and sequenced. Live fishes of *A. lineatus* were collected from Bay of Bengal at Rameswaram (9°15′00″N, 79°20′00″E), Tamil Nadu, India. Specimens were identified, tagged (Voucher No: AE02) and deposited in the laboratory museum. The mitochondrial genome (GenBank accession no EU273284) was a circular molecule of 16,532 bp in length and contained 2 rRNA genes, 22 distinct tRNA genes, 13 protein-coding genes (PCGs) and a control region. Most of the peptide-coding genes are encoded in the heavy strand (H) punctuated by one or more alternate tRNA genes except ND6 and eight tRNA genes similar to other vertebrates (Zardoya et al. 1995; Inoue et al. 2000). The intergenic spacers ranged from 1 to 35 nucleotides.

The overall nucleotide composition was 29.84% A, 28.07% C, 26.60% T and 15.49% G with an A + T content of 56.44%, and hence the overall C/G ratio is 1.81.The overall base compositions of 13 protein-coding genes are 11,433 bp (69.16% of whole mt genome). ATG was the initiation codon for all PCGs. Five protein-coding genes (ND1, ATPase8, ND4L, ND5 and ND6) ends with a complete termination codon TAA whereas ND2, COI, ATPase 6 and COlll ends with TA and COll, ND3, ND4 and Cyt b ends with a T residue. Such immature stop codon is completed via post-transcriptional polyadenylation (Ojala et al. 1981). The two 12S and 16S rRNA genes were 950 and 1689 bp long with and an A + T content of 52.21% and 55.42%, respectively.

Twenty-two tRNA genes are interspersed between the rRNA and protein-coding genes. Most of the tRNA genes are encoded in the H-strand with a size ranging from 65 to 76 bp. The well-conserved tRNA clusters (IQM, WANCY and HSL) were also observed. The noncoding control region localized between tRNA^Pro^ and tRNA^Phe^ genes was 852 bp long containing eight termination-associated motif sequence (TAS) and three conserved sequence blocks (CSB-1, CSB-2 and CSB-3). The origin of replication (OL) of 50 bp length located in the WANCY region between tRNA^Asn^ and tRNA^Cys^ has the potential to form a stable stemloop secondary structure with 13 paired nucleotides in the stem and 13 nucleotides in the loop with a conserved motif (5'-GGCCG-3') at the base of the stem loop structure.

The MP tree was constructed using MEGA 6 software (Tamura et al. 2013) for 17 most closely related species of Perciformes based on the concatenated nucleotide sequences of 12 mitochondrial encoded H-strand protein genes. It showed that *A. lineatus* was clustered together with other Acanthuroidei fishes forming monophyletic (Figure 1).

**Figure 1.**
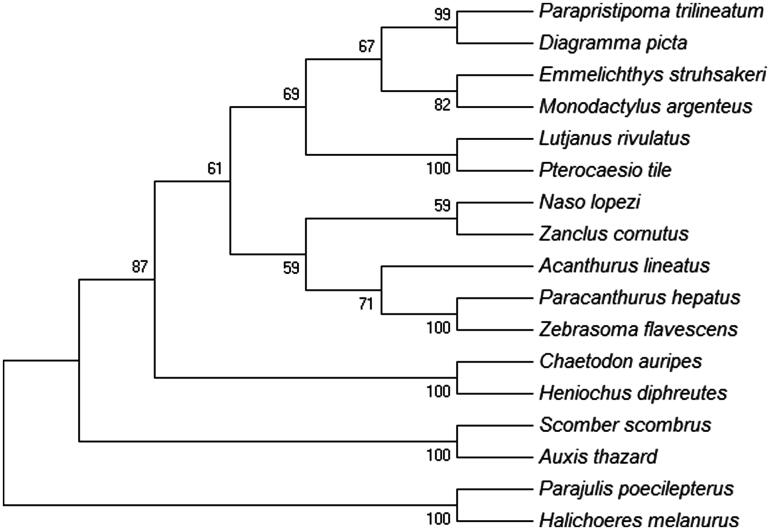
MP tree was generated using the MEGA 6 program. Number on the nodes correspond to bootstrap values based on 1000 replicates. The most parsimonious tree with length =22,730. The consistency index is (0.379542), the retention index is (0.264109), and the composite index is 0.110326 (0.100240) for all sites and parsimony-informative sites (in parentheses). There were a total of 10,846 positions in the final dataset. The accession numbers of the species are *Parajulis poecilepterus* (EF192032.2), *Halichoeres melanurus* (AP006018.1), *Chaetodon auripes* (AP006004.1), *Heniochus diphreutes* (AP006005.1), *Scomber scombrus* (AB120717.1), *Auxis thazard* (AB105447.1), *Naso lopezi* (AP009163.1), *Zanclus cornutus* (AP009162.1), *Acanthurus lineatus* (EU273284.2), *Paracanthurus hepatus* (KT826539.1), *Zebrasoma flavescens* (AP006032.1), *Lutjanus rivulatus* (AP006000.1), *Pterocaesio tile* (AP004447.1), *Parapristipoma trilineatum* (AP009168.1), *Diagramma picta* (AP009167.1), *Emmelichthys struhsakeri* (AP004446.1) and *Monodactylus argenteus* (AP009169.1).
